# Shorter Duration of Post-Operative Antibiotics for Cecal Ligation and Puncture Does Not Increase Inflammation or Mortality

**DOI:** 10.1371/journal.pone.0163005

**Published:** 2016-09-26

**Authors:** Kendra N. Iskander, Max Vaickus, Elizabeth R. Duffy, Daniel G. Remick

**Affiliations:** 1 Department of Surgery, Boston University Medical Center, 88 East Newton Street, C 515, Boston, MA, 02118, United States of America; 2 Department of Pathology and Laboratory Medicine, Boston University School of Medicine, 670 Albany Street, Room 441, Boston, MA, 02118, United States of America; Universidade de Sao Paulo Faculdade de Medicina, BRAZIL

## Abstract

Antimicrobial therapy for sepsis has beneficial effects, but prolonged use fosters emergence of resistant microorganisms, increases cost, and secondary infections. We tested whether 3 days versus 5 days of antibiotics in the murine model of cecal ligation and puncture (CLP) negatively influences outcomes. Following CLP mice were randomized to receive the antibiotic imipenem-cilastatin (25mg/kg) in dextrose 5% in Lactated Ringer’s solution every 12 hours for either three or five days. Serial monitoring over 28 days included body weight, temperature, pulse oximetry, and facial vein sampling for hematological analysis and glucose. A separate group of mice were euthanized on post-CLP day 5 to measure cytokines and peritoneal bacterial counts. The first study examined no antimicrobial therapy and demonstrated that antibiotics significantly improved survival compared to fluids only (p = 0.004). We next tested imipenem-cilastatin therapy for 3 days versus 5 days. Body weight, temperature, glucose, and pulse oximetry measurements remained generally consistent between both groups as did the hematological profile. Pro-inflammatory plasma cytokines were comparable between both groups for IL-6, IL-1β, MIP-2 and anti-inflammatory cytokines IL-10, and TNF SRI. At 5 days post-CLP, i.e. 2 days after the termination of antibiotics in the 3 day group, there were no differences in the number of peritoneal bacteria. Importantly, shortening the course of antibiotics by 40% (from 5 days to 3 days) did not decrease survival. Our results indicate that reducing the duration of broad-spectrum antibiotics in murine sepsis did not increase inflammation or mortality.

## Introduction

Sepsis, a systemic inflammatory response to a severe infection, results in thousands of hospital admissions per year with an estimated cost between $16 to $24 billion annually in the United States [1]. The rate of sepsis hospitalizations in the US has increased, but the length of hospital stay and mortality rate has decreased [2]. These declines in hospital stays and death rates suggest an improvement in sepsis treatment which parallels clinical advancements. However, the usual treatment approach of fluid resuscitation, antibiotics, and end organ supportive care is still poorly tailored to the complexities of sepsis and the myriad of complications that can occur [3].

Antibiotic therapy has a central role in the clinical treatment of sepsis. Prior to obtaining definitive bacterial cultures and sensitivities, empiric antimicrobial treatment is routinely initiated in septic patients. Administration of broad-spectrum antimicrobials are initially used for this life-threatening condition to increase the likelihood that early treatment will be effective against the infectious pathogen(s) [4]. The risk of death from sepsis is significantly greater if the original antibiotic regimen does not have adequate antimicrobial coverage and is not delivered in a timely manner [5]. Converting to an appropriate drug after receiving culture results does not diminish this risk [5, 6]. Antibiotic coverage may be narrowed once specific microbes are identified. However, this initial-broad spectrum approach and the prolonged use of antibiotics are associated with adverse effects such as microbial resistance, increased cost, and secondary infections such as *Clostridium difficile* colitis. Shortening the duration of antimicrobial therapy will not only decrease costs but also lower chances of an infection with resistant organisms like Candida species, vancomycin resistant *Enterococcus faecium*, or *Clostridium difficile*.

The cecal ligation and puncture (CLP) murine model of sepsis has been widely used to study sepsis. The CLP model produces a broad systemic response due to the presence of gram negative and gram positive flora from the cecum, including anaerobic and aerobic bacteria, as well as necrotic tissue from the ischemic cecum [7]. Prior antibiotic protocols were effective in eliminating positive blood cultures following CLP [8] and have been routinely administered for 5 days post-operatively [8–10]. It is unclear whether a shorter course of antibiotics in the murine model of CLP will influence outcomes. We investigated whether 3 days of imipenem-cilastatin, rather than the traditional 5 days, will alter physiological parameters, inflammatory biomarkers, and survival.

## Materials and Methods

### Animals

Female ICR (CD-1) outbred mice (Harlan Laboratories, Inc.) were subjects in the experiment. Mice were housed in a temperature controlled room set on a diurnal 12-hour light-dark cycle and allowed to acclimate for at least 72 hours before intervention. Animals were provided food and water *ad libitum* for the entire study. The experiments were approved by Boston University Animal Care and Use Committee. Using only female mice for the study is a limitation.

### Sepsis model

Cecal ligation and puncture was conducted according to our laboratory protocol [11, 12] an updated version of the original model [13]. Using 3% isoflurane for anesthesia, the fur on the abdomen was trimmed with an electric clipper to expose the skin. The abdomen was prepped with chlorhexidine solution (Bimeda, Inc., Oakbrook Terrace, IL) and a 2 cm midline abdominal incision was made through the skin with subsequent opening of the underlying peritoneum. The cecum was externalized and ligated with 4.0 silk suture followed by 16 gauge needle double puncture to induce a polymicrobial septic insult. The peritoneum was closed with interrupted 4.0 silk sutures and the skin with tissue adhesive. Animal suffering was reduced with buprenorphine (0.05mg/kg), given subcutaneously for analgesia every 12 hours during the first 48 hours after CLP, for a total of 4 doses. The initial dose was given in 1mL of warmed normal saline immediately following the surgical procedure. Mice were weighed daily, monitored twice per day and humane endpoints used to determine if the mice were moribund and met criteria to be euthanized [14]. These criteria included inability to stand, agonal breathing, decreased body temperature to less than 30°C for more than 6 hours or excessive loss of body weight. Mice meeting criteria were euthanized by cervical dislocation under isoflurane anesthesia with verification of death by removal of vital organs.

### Antibiotic regimen

A review of the CLP literature from 2015 to 2016 showed a range of duration of antibiotic use from a single injection at the time of surgery to providing up to 5 days of antibiotics (Table 1). Out of the 10 studies, 9 used a carbapenem based antibiotic, a regimen that has been shown to be effective in CLP [8]. None of these studies compared the efficacy of length of time of antibiotic treatment. Since we have routinely used 5 days of treatment [15–17] we designed a study to directly compare if a 40% reduction in the duration of antibiotic therapy would have a worse outcome. Mice were randomized to receive injections of the broad-spectrum imipenem-cilastatin (Merck, West Point, PA) in dextrose 5% in Lactated Ringer’s solution (D_5_LR) for either the standard 5 days or the experimental 3-day course. Antibiotic treatment began 2 hours after CLP with imipenem-cilastatin (25 mg/kg) and continued every 12 hours for the first 5 days (10 doses) or the first 3 days (6 doses). The antibiotic regimen was followed until murine death or completion of the protocol.

**Table 1:** Antibiotic therapy used in murine cecal ligation and puncture studies.

Antibiotic	Duration (reference)
Imipenem	Single dose [18]
Imipenem	Single dose [19]
Imipenem	Single dose [20]
Imipenem	Single dose [21]
Ertapenem	Single dose [22]
Ceftriaxone + metronidazole	1.5 days [23]
Ertapenem	3 days [24]
Ertapenem	3 days [25]
Ertapenem	3 days [26]
Imipenem	5 days [27]

Multiple studies show that the length of antibiotic treatment varies greatly, although virtually all studies used a carbapenem based antibiotic. Literature reviewed April, 2016.

In a separate experiment to demonstrate the effects of antibiotics on mortality in sepsis, a different group of mice received the antibiotic regimen twice daily for 5 days compared to mice that were given fluid only (D5W in Lactated Ringers) at the corresponding administration times also for 5 days. Mice were followed for survival over 28 days.

### Physiological monitoring of body weight, temperature, and oxygen saturation

Body weights were recorded at baseline (prior to surgery) and then measured serially until time of death or post-CLP day 28. Murine body temperature was obtained non-invasively by infrared probe (Infrared Thermometer, Fisher Scientific, Pittsburgh, PA) directed at the abdomen in the lower right quadrant distant from the midline incision site. Pulse oximetry with a collar sensor (STARR Life Sciences Corp, Mouse Ox, Oakmont, PA) was used to measure oxygen saturation.

### Blood and peritoneal sampling

For serial sampling of live mice over 28 days, the facial vein (*vena submandibularis)* was punctured with a 23 gauge needle and 20 μL of blood was removed with a pipette tip rinsed with EDTA (169 mM tripotassium salt). Samples were immediately diluted 1:10 in phosphate-buffered saline with a 1:50 dilution of EDTA. Blood was centrifuged (5 min/1000 G at 4°C), the diluted plasma removed, and the cell pellet used for hematological analysis. At the time of facial vein puncture in subsequent experiments, an additional 4 μL of blood was drawn into a different pipette and used directly for blood glucose measurements.

In the mice that were harvested on post-operative day 5, cardiac puncture was conducted percutaneously using a 25 gauge needle and blood was collected in a syringe and mixed with 50 uL of 169mM EDTA and plasma stored at -80°C for cytokine analysis. After euthanasia, the peritoneal cavity was opened and lavaged with a 1 mL aliquot of warm HBSS (Mediatech, Herndon, VA). A 100 uL portion of the 1 mL lavage was used directly to plate serial dilutions of peritoneal fluid for bacteria cultures. The remaining lavage fluid was centrifuged and the supernatant frozen at -80°C for subsequent detection of cytokine levels by ELISA.

### Hematology

Following facial blood collection, the cell pellet was immediately re-suspended in 480 μL of Hemavet solution (CDC Technologies, Oxford, CT). A complete blood count including differential was performed in a Hemavet 1500 (CDC Technologies, Oxford, CT).

### Glucose measurement

Circulating glucose concentrations were determined directly in undiluted blood using the TRUEtrack glucometer (McKesson, Richmond, VA).

### Bacterial cultures

For bacterial culturing, 100 μL of fluid obtained immediately after peritoneal lavage was diluted with HBSS 1:10–1:10^4^, and 20 μL of each dilution was aliquoted onto 5% sheep blood agar plates (Fisher Scientific, Pittsburgh, PA). The plates were incubated at 37°C in either aerobic or anaerobic conditions for 24 hours, and the number of colony forming units (CFUs) was quantified.

### Enzyme-linked immunosorbent assay (ELISA)

An aliquot of plasma was diluted 1:10 and used to determine the pro and anti-inflammatory cytokine concentration by ELISA as previously described [28]. The supernatant from the peritoneal lavage fluid was diluted 1:2 for IL-6 and MIP-2 and 1:1 for the remaining cytokines detected by ELISA. A panel of 13 cytokines, both pro- and anti-inflammatory was chosen since they have been shown to have a role in the course of sepsis [29].

### Statistical analysis

Data were analyzed with Prism 5 (GraphPad Software). Results were reported as mean ± SEM unless otherwise noted. Statistical significance for survival curves was determined using a Log-rank (Mantel-Cox) Test. For repeated measurements of physiological and hematological data over a 28 day period, a two-way ANOVA followed by a Bonferroni post hoc correction was used to measure statistical significance. A repeated measures ANOVA could not be used as this was a longitudinal measurement of the same mice. Since some mice died during the observation period, our total number of mice per group would be reduced if analysis was limited to survivors. Cytokine and peritoneal CFU levels were analyzed using an unpaired two-tailed Student’s t-test, since these data were normally distributed.

## Results

### Antibiotic + fluid resuscitation

**Fig 1:**
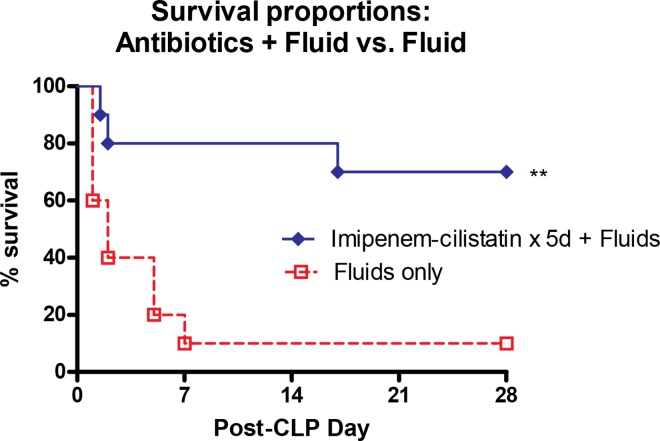
Antibiotics+Fluids vs. Fluids alone survival. Mice receiving antibiotics plus fluid resuscitation versus fluids only have a significantly better survival following CLP. Mice received 5 days of antibiotic therapy post-cecal ligation and puncture (CLP) treatment with 1ml D_5_LR and the broad spectrum antibiotic imipenem-cilastatin (25 mg/kg) compared to septic mice receiving just 1 ml of D_5_LR for 5 days post-CLP. N = 7–10 mice per group. **p <0.01 comparing the two groups.

The first experiment demonstrated that a 5 day treatment protocol post-CLP with imipenem-cilastatin and fluid resuscitation compared to fluids alone significantly improved survival (Fig 1), confirming the clinical studies that prompt and appropriate antibiotic therapy improves survival [6].

### Physiological monitoring

**Fig 2:**
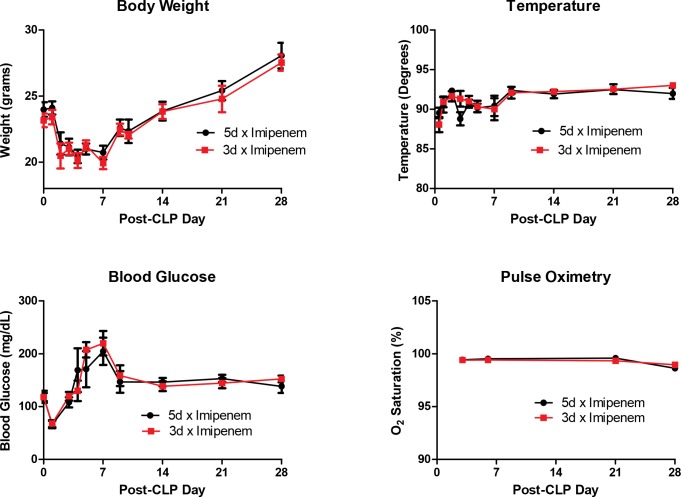
Physiological Parameters. There were no physiological differences between mice receiving 5 days of imipenem-cilastatin treatment versus 3 days of treatment following CLP. Data expressed as mean ± SEM (n = 16–22 mice per group for body weight, temperature, and blood glucose measurement. N = 6 mice per group for pulse oximetry).

Body weight, temperature, blood glucose, and O_2_ saturation remained comparable at all time points to 28 days after CLP, suggesting a similar physiological response to 5 days of antibiotics versus 3 days (Fig 2). As previously reported, body weight decreases after CLP and then recovers [14], and a stress hyperglycemia occurs in the first week of sepsis [17]. Notably, at 5 days post-CLP, there is no statistical difference between treatment groups in body weight, body temperature, or blood glucose (Table 2).

**Table 2:** Physiological and glucose measurements 5 days post-CLP.

5 days post-CLP		
	5d x Imipenem	3d x Imipenem
Body Weight (Grams)	21.0 ± 0.4	21.0 ± 0.4
Body Temp (C)	30 ± 0.3	30 ± 0.4
Blood Glucose (mg/dl)	171 ± 34	207 ± 14

There are no significant differences between body weight, temperature, and blood glucose measurements on day 5 post-CLP. Data expressed as mean ± SEM, n = 14 mice per group for weight and temperature, n = 6 for glucose.

### Hematologic changes

**Fig 3:**
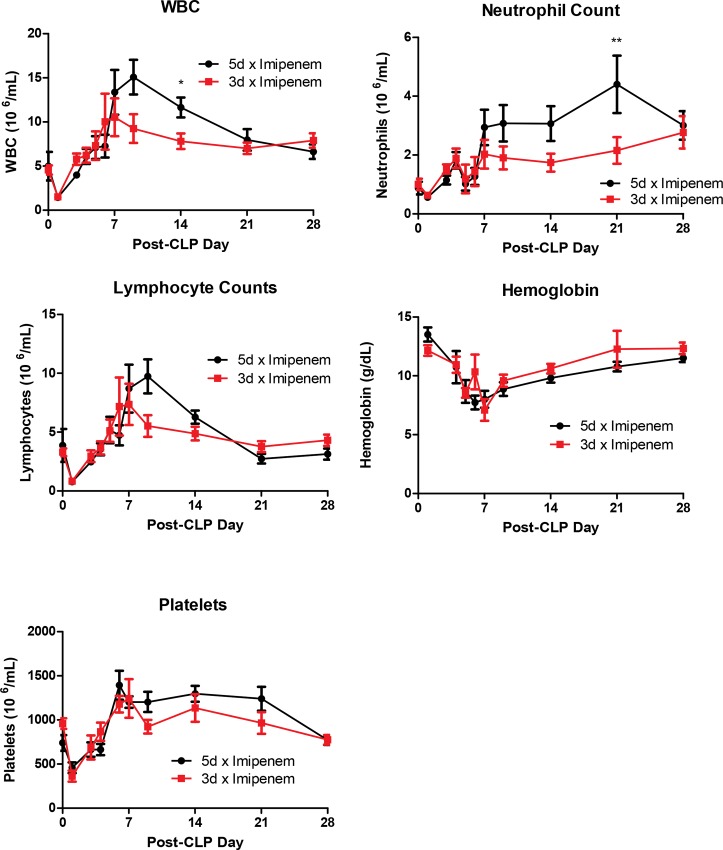
Hematologic changes. The shorter 3 day antibiotic treatment did not significantly change peripheral blood measurements in septic mice compared to 5 days of treatment. Five day treatment mice showed elevations of WBC, neutrophil, and lymphocyte counts starting at day 7 which normalized by day 28. Five day treatment had significantly higher WBC and neutrophil counts only at 14 and 21 days post-CLP, respectively. The WBC and neutrophil count remained elevated at day 28 compared to the pre-CLP values, probably due to the ongoing stress of the infection. All other hematologic changes returned to pre-CLP (day 0) levels by day 28. Data expressed as mean ± SEM, n = 16–18 per group, *p < .05 **p < .01.

Following CLP-induced sepsis, there was an initial drop in circulating leukocytes which then rebounded above baseline levels within the first week (Fig 3). The decrease in lymphocytes due to apoptosis [30] also results in the overall decline in the white count and the drop in neutrophils has also been reported following CLP [31]. Minor hematologic changes occurred at discrete time points post-CLP which were probably of little biological relevance. Hemoglobin and platelet levels were not significantly different between the groups up to 28 days post-CLP. Elevated numbers of white blood cells persisted until day 28 and the lymphocyte count remained elevated until day 21. However, the only significant differences by Bonferroni post test were at day 14 for white blood cells and day 21 for neutrophils for 5-day treatment mice (Fig 3). It is possible that the increased neutrophil count reflected a secondary infection in the 5 day treatment regimen, but this is unlikely since there was no change in temperature or body weight typically observed in an infection.

### Plasma cytokine profile

Biomarkers measured within the first 24 hours after CLP predict outcome, however, both the 3-day and 5-day antibiotic groups would have received identical therapy within the first 24 hours so there should not be any differences at this time point. As anticipated, at day 1 post-CLP there was no significant difference in circulating levels of 13 different cytokine and cytokine inhibitors (Table 3) between the 5-day or 3-day treatment protocols. The only cytokines showing a significant difference were RANTES and MCP1 with elevated levels of each cytokine in the 5-day treatment mice compared to 3-day treatment mice, which can most likely be attributed to natural variation as at 1 day post-CLP, the groups are receiving the same treatment (Table 3).

**Table 3:** Pro-inflammatory and anti-inflammatory cytokines at day 1 post-CLP.

Day 1 post-CLP		
	5d Imipenem (pg/ml)	3d Imipenem (pg/ml)
IL-1B	753 ± 440	620 ± 179
IL-2	872 ± 263	732 ± 299
IL-6	1396 ± 353	1081 ± 230
IL-10	928 ± 307	868 ± 137
IL-12p70	225 ± 52	241 ± 39
IL-13	3101 ± 1843	2284 ± 1214
IFN g	215 ± 60	210 ± 48
MIP-2	357 ± 103	332 ± 109
MIP1a	1191 ± 544	1305 ± 485
*RANTES	12078 ± 5071	8603 ± 1417
*MCP1	2466 ± 961	1264 ± 232
TNF sr1	162 ± 41	145 ± 30
TNF sr2	67 ± 24	49 ± 8
IL-17	89 ± 46	201 ± 27
ICAM	92928 ± 5828	95476 ± 4547

Five day treatment mice had significantly higher levels of RANTES and MCP1 versus 3 day treatment mice, most likely due to natural variation. Data expressed as mean ± SEM, n = 3–5 mice per group.

*p < .05

Another panel of both pro-inflammatory and anti-inflammatory cytokines was measured at day 5 post-CLP, i.e. 2 days after the termination of antibiotic therapy in the 3 day treatment group. At day 5 post-CLP there were essentially no differences in multiple cytokines and cytokine inhibitors. MCP1 was the only cytokine with a significant difference at day 5 post-CLP, with the 3-day treatment mice showing higher levels (Table 4).

**Table 4:** Pro-inflammatory and Anti-inflammatory Cytokines 5 days post-CLP.

Day 5 post-CLP		
	5d x Imipenem (pg/ml)	3d x Imipenem (pg/ml)
IL-1B	130 ± 51	112 ± 46
IL-2	562 ± 472	600 ± 310
IL-6	180 ± 27	252 ± 77
IL-10	170 ± 30	264 ± 86
IL-12p70	119 ± 29	93 ± 36
IL-13	904 ± 249	511 ± 217
IFN g	98 ± 27	107 ± 35
MIP-2	66 ± 17	88 ± 19
**MCP1	4 ± 1	45 ± 42
TNF sr1	88 ± 8	100 ± 12
TNF sr2	46 ± 23	40 ± 12
ICAM	64626 ± 12500	61288 ± 7066

Plasma cytokine levels on day 5 post-CLP sampled from the facial vein and detected by ELISA. There were no significant differences between the 5 day versus 3 day treatment groups except MCP1, which was significantly higher in the 3 day treatment group. Data expressed as mean ± SEM, n = 3–5 mice per group.

**p < .01

### Peritoneal bacterial load

**Fig 4:**
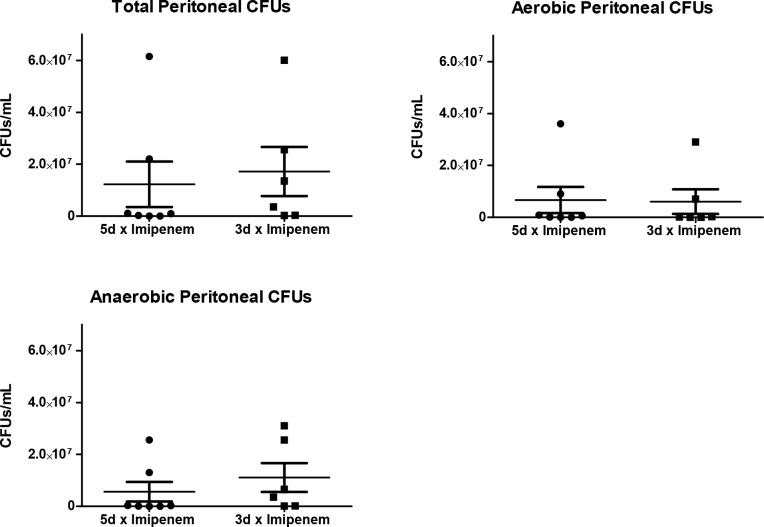
Peritoneal Lavage bacterial CFUs day 5 post-CLP. Mice were euthanized at day 5 post-CLP and the peritoneum lavaged and cultured. There were no significant differences in the number of bacterial colony forming units. Individual data points are show as well as the mean ± SEM, n = 6–7 mice per group.

A significant question is whether the shorter antibiotic therapy would result in increased growth of bacteria. On day 5 post-CLP, peritoneal lavage fluid was tested for total CFUs, aerobic and anaerobic CFUs. While the 3-day treatment group showed slightly higher total and anaerobic CFU levels, these numbers were not significantly different (Fig 4).

### Mortality

**Fig 5:**
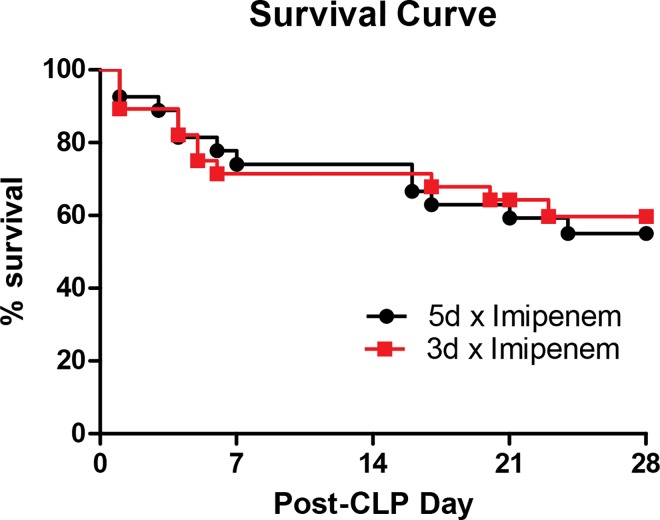
Survival Proportions. There was no significant survival difference between the 5 days versus 3 days treatment group following CLP. N = 27–28 mice per group. There is also no statistically significant difference between the survival of the 5 day mice in this group and the comparable group in Fig 1.

The major outcome of sepsis studies, especially in the clinical literature, is the 28-day all-cause mortality. There was no significant difference in survival between 5-day and 3-day treatment mice (Fig 5, p = 0.78). The mortality rate for both treatment groups post-CLP remained around 50%. In the 3-day imipenem group, 11 out of 28 died within 28 days, while 12 out of 27 mice in the 5-day imipenem group died within 28 days. Using a Log-rank (Mantel-Cox) Test, we compared the mortality of the ten mice in the 5-day treatment group for Fig 1 to the mortality of the 28 mice in the 5-day treatment group used for Fig 5 and found no statistical difference between their mortality (P = 0.49) highlighting the reproducibility of our CLP model. This comparison was done to address the National Institutes of Health initiative on data reproducibility [32].

## Discussion

Antibiotics are a mainstay for treating life-threatening conditions that arise among critically ill patients. However, their profligate use encourages the development of antibiotic resistant organisms such as *Pseudomonas aeruginosa*, carbapenem-resistant *Enterobacteriaceae*, and methicillin-resistant *Staphylococcus aureus*, in addition to fostering *C*. *difficile* infections [33]. The spread of these multi-drug resistant organisms has increased efforts to reevaluate the standard approach to treatment. It has been suggested that the usual course of antibiotics, especially in sepsis, has uncertain benefit and could be reduced, decreasing the risk of developing antibiotic resistant organisms [34]. Liberal antibiotic usage is thought to be the leading cause of antibiotic resistance around the world. Despite this evidence, global antibiotic consumption has increased over the last decade, highlighting the importance of antibiotic stewardship studies and programs [35]. However, few studies are able to safely and thoroughly measure the effect that a shortened antibiotic treatment has on physiological, hematological, and survival effects in a clinical setting. Using our CLP-induced murine model of sepsis, we analyzed the differences between a 3-day antibiotic regimen and a 5-day treatment. Our study showed no significant difference in survival or bacterial CFUs between a shorter duration of antibiotics.

Biomarkers such as procalcitonin (PCT) have been proposed to help guide the difficult clinical decision of when to stop antibiotics. A retrospective review found that PCT-guided therapy is linked with an average reduction of two days of antibiotic use with no adverse effects on clinical outcomes [36]. While still debated, these studies suggest PCT’s utility as a gauge to appropriately shorten antibiotic usage. Another biomarker with potential use for antibiotic stewardship is C-reactive protein (CRP), which could provide similar benefits as PCT in terms of antibiotic discontinuation [37]. While CRP holds promise as an affordable and simple biomarker to measure, more research is needed to determine its value in regards to antibiotic stewardship [38]. The presence of leukocytosis or fever serves as a simpler, widely available predictor of appropriate antibiotic termination. In one study of patients who underwent surgical drainge for a septic infection, 7 of 21 patients who had leukocytosis when their antibiotic regimens were stopped after operation developed intra-abdominal infections compared to no intra-abdominal infections in 30 patients who had normal leukocyte levels when antibiotics were stopped [39]. In our study mice in both groups had leukocytosis and normal body temperature on day three post-CLP suggesting that more specific parameters would have greater predictive power.

Another biomarker which could help in antibiotic stewardship is IL-6. Using our murine sepsis model, we found that IL-6 effectively predicted mortality [31]. In this study, septic mice were sampled for plasma measurements of IL-6 at 6 hours and when levels exceeded 2000 pg/ml, mortality within the first 3 days of surgery could be predicted with a specificity of 97% and a sensitivity of 58%. However, an early spike of cytokines in murine sepsis is not always present as our lab has shown that some mice who die in the chronic phase of sepsis (6–28 days post-CLP) do not show an early spike in cytokine levels [40]. Instead, mice succumbing to chronic-phase sepsis often exhibited a significant pre-lethal increase in various pro-inflammatory (TNF-α, MIP-2, MCP-1) and anti-inflammatory (IL-1ra, TNF-SR1) cytokines compared to healthy mice [40]. The mortalities occurring in the chronic phase fall outside the 5 day post-CLP window when antibiotics are administered, complicating the use of an early cytokine level as a biomarker to curtail antibiotic use.

Antibiotic stewardship is an approach meant to limit excessive use of antibiotics, but in the clinical setting, quick and appropriate antibiotic administration is crucial. However, the correct duration may be shorter than previously believed. An examination of patients with hospital-acquired pneumonia given a shortened 7 to 8 day course of antibiotic therapy saw no significant difference in risk of adverse outcome compared to patients on a typical course of 10 to 15 days of antibiotics [41]. A study examining community-acquired intraabdominal infection found no significant difference in clinical response and bacteriological efficacy between patients given the standard 5-day duration of ertapenem versus 3-day duration [42]. Our study, which sought to determine the result of reducing the duration of broad-spectrum antibiotics in intra-abdominal sepsis in a murine model post-CLP, paralleled these clinical studies. We found that a decreased duration of antibiotics does not increase inflammatory cytokines, bacterial load, or mortality. Additionally, there are no differences in any physiological parameters, peripheral blood measurements, or body weight between treatment groups. The ability to administer fewer days of antibiotics, without harmful effects, is reflected in current clinical studies and may alter the traditional CLP model to be more cost-saving and clinically relevant.

Conventional thinking recommends that a course of antibiotics needs to be completed to prevent the development of resistant organisms that may occur with abbreviated use [43]. This concept is being reassessed as studies have suggested that a shorter antibiotic treatment has no effect on outcome or development of resistant organisms [42, 44]. An important clinical study on the treatment of peritonitis (STOP IT) showed that reducing the days of antibiotic usage by 50% did not increase mortality [45]. This reduction was similar to our 40% reduction in the days of antibiotic use, and similar to our study there was no evidence of increased mortality with a shorter duration of antibiotic use.

There are some limitations to the current study. First, we only tested 3 days versus 5 days of therapy. It is possible that an even shorter duration of antibiotic therapy would be just as efficacious. Second, only female mice were used in these studies and future investigations will need to examine if similar observations may be found in male mice. Third, this is a murine model of sepsis and not actual clinical data.

The use of mice to study inflammatory responses including sepsis is controversial. A recent study compared the genomic responses in mice and humans and found few changes were similar [46]. However, a subsequent re-analysis of the same genomic data reached the conclusion that the genomic changes were quite similar [47]. Additionally, a recent review listed 26 separate publications where murine studies accurately predicted subsequent human studies [48]. Among these studies were publications showing that antibodies to TNF would not improve sepsis survival which was published 3 years before the human studies demonstrated that TNF inhibitors were not effective [49, 50]. Properly conducted murine studies do have the potential to appropriately direct future human studies and provide important insights.
